# Efficient Guided Wave Modelling for Tomographic Corrosion Mapping via One-Way Wavefield Extrapolation

**DOI:** 10.3390/s24123750

**Published:** 2024-06-09

**Authors:** Emiel Hassefras, Arno Volker, Martin Verweij

**Affiliations:** 1 Acoustic Sensor and Sonar Systems, Department of Acoustic & Underwater Warfare, Netherlands Organisation for Applied Scientific Research (TNO), 2597 AK Den Haag, The Netherlands; arno.volker@tno.nl; 2 Laboratory of Medical Imaging, Department of Imaging Physics, Delft University of Technology, 2628 CJ Delft, The Netherlands; m.d.verweij@tudelft.nl

**Keywords:** corrosion monitoring, extrapolation operators, acoustic formulation, tomography

## Abstract

Mapping corrosion depths along pipeline sections using guided-wave-based tomographic methods is a challenging task. Accurate defect sizing depends heavily on the precision of the forward model in guided wave tomography. This model is fitted to measured data using inversion techniques. This study evaluates the effectiveness of a recursive extrapolation scheme for tomography applications and full waveform inversion. It employs a table-driven approach, with precomputed extrapolation operators stored across a spectrum of wavenumbers. This enables fast modelling for extensive pipe sections, approaching the speed of ray tracing while accurately handling complex velocity models within the full frequency band. This ensures an accurate representation of diffraction phenomena. The study examines the assumptions underlying the extrapolation approach, namely, the negligible reflection and conversion of modes at defects. In our tomography approach, we intend to use multiple wave modes—$A_{0}$, $S_{0}$, and $SH_{1}$—and helical paths. The acoustic extrapolation method is validated through numerical studies for different wave modes, solving the 3D elastodynamic wave equation. Comparison with an experimentally measured single-mode wavefield from an aluminium plate with an artificial defect reveals good agreement.

## 1. Introduction

Accurately estimating corrosion rates in pipe networks is crucial for safe operations within the petrochemical industry. However, the traditional method of inspecting large structures to detect corrosion using conventional ultrasonic bulk wave techniques is time consuming. Fortunately, ultrasonic-guided waves have emerged as a viable alternative. These waves can be generated at a single point on the structure and propagate over relatively long distances with minimal attenuation [1,2]. Guided wave testing can be a complex process due to the presence of several potential wave modes, most of which are dispersive. This means that the velocity of these waves is dependent on the frequency chosen and the local thickness of the waveguide [3]. In tomography, we exploit this dispersive property to our advantage. Corrosion-induced variations in wall thickness lead to measurable differences in the phase and amplitude of the signals received [4,5,6,7,8,9]. Subsequently, these measurements are compared to a forward model, where the local wall thickness is iteratively refined to minimise the difference between the modelled and observed signals.

**Figure 1:**
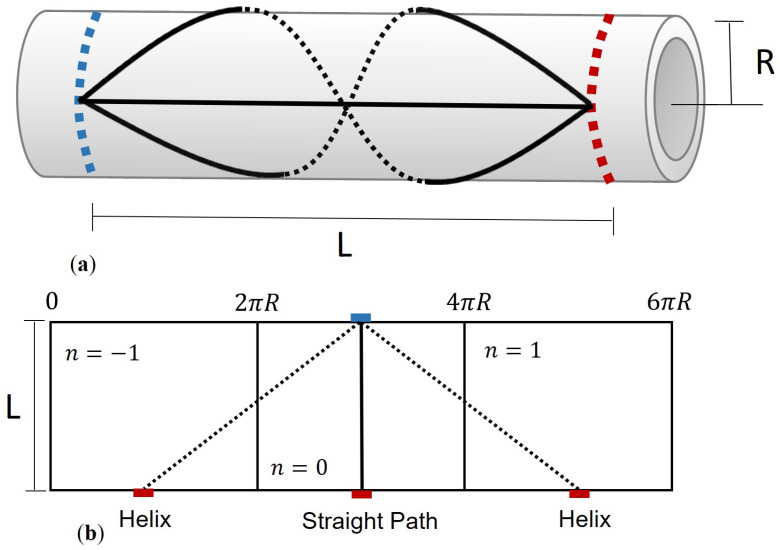
Unwrapping of a pipe section. (**a**) Three wavepaths along a pipe section originating from the same source and arriving at the same receiver. (**b**) Unwrapped geometry displaying the direct path and two helical modes with replication values of *n* = [−1 0 1].

Guided wave tomography (GWT) has primarily been applied to plates, enabling comprehensive defect illumination from various angles using circular arrays. In practical implementations, velocity inversion algorithms are employed to estimate velocities and locate defects, often relying on ray theory [10,11]. While this approach provides valuable insights, its applicability may be limited due to neglecting diffraction effects. Alternatively, to improve resolution, one can use either diffraction tomography [12] or a combination of both diffraction and ray-based tomography [5,8,13,14]. Beamforming algorithms can then be used to image the defects. Similarly, when sections of straight pipes are treated as flat plates under the assumption that the wall thickness is much smaller than the pipe radius [15,16,17,18], tomography can be applied to a pipe section delimited by two rings of ultrasonic transducers, as depicted in Figure 1 [4,19,20,21].

However, due to the limited-view geometry of the source–receiver pairs for pipes, the problem becomes highly ill posed. Addressing this non-uniqueness is crucial, and it can be achieved through various methods such as smarter parameterisation for travel time tomography [6,22], incorporation of additional information by considering more helical paths for illumination under higher angles [23], or leveraging all waveform information in Full Waveform Inversion (FWI), as commonly applied in exploration seismology [24,25,26]. In recent years, FWI techniques have been adopted in guided wave tomography, both in full-aperture scenarios [27,28] and limited-view applications [29,30]. The latter implementation still suffers from non-uniqueness, and achieving a reasonable thickness reconstruction requires employing extremely high angles with a long sensor array placed close to the defect. Additionally, strong regularisation must be applied, with the risk of over-smoothing. Reconstructing a relatively simple defect shape like a Gaussian profile presents no significant challenges. Yet, when dealing with more complex defects, like a deep pit next to moderate wall thickness loss, our objective is to restrict this form of regularisation. This is to avoid smearing the overall wall thickness loss across the entire defect area.

In our research, we attempt to address this problem differently. Alongside employing a phase and amplitude approach similar to an FWI implementation, complemented by multiple helical paths, our intention is to establish a misfit through a joint inversion for multiple wave modes. However, as a result, a significant challenge arises in selecting the appropriate forward model to describe wave propagation behaviour. Ideally, a model should accurately portray relevant physics, be computationally efficient, and be highly sensitive to target property changes. As we intend to propagate multiple wave modes in parallel during the inversion process, prioritising computational efficiency becomes essential. This is especially true for parameterised guided wave tomography (GWT) [7,30], where defining numerous parameter points to describe complex defect shapes is necessary. Additionally, the iterative nature of inversion also necessitates careful consideration of the computational complexity of the forward solver.

Although the 3D elastodynamic wave equation provides precise descriptions of guided wave interactions with defects, their computational demands often render them impractical for real-world applications. As a solution, many current GWT implementations employ 2D acoustic forward models [27,28,31,32]. These models simplify complexity by representing the wavefield through a single scalar pressure field. This approach relies on Lamb wave phase velocity dispersion curves, where the frequency–thickness product is important. Defects affect guided wave propagation similarly to perturbations experienced by acoustic pressure waves in regions with nonuniform sound speeds. This establishes a direct correlation between wall thickness in a plate and sound speed in an acoustic model.

In this study, we develop an alternative, faster acoustic forward model, specifically, one that describes one-way wave propagation. The efficacy of this forward model has been previously established in seismic applications [33,34,35,36,37,38,39,40,41]. Protzgen et al. employed wavefield extrapolation in the space–frequency domain (FX) for conventional ultrasonic imaging [42]. Subsequently, the recursive scheme was utilised for corrosion mapping, leveraging phase information [6]. Although the method can be extended for the use of anisotropic materials [7], our current focus is solely on isotropic metals. In our forthcoming inversion algorithm, we aim to incorporate both phase and amplitude information to define the misfit function. Consequently, this paper seeks to extend the validation of the recursive extrapolation model by assessing its accuracy in the presence of realistic corrosion defects. The realistic complex defects are provided by the industry and have been studied by other research groups [8,13]. They primarily exhibit extensive corrosion larger than 10 cm but with significant spatial variation due to local pitting within the defect. This validation study comprises two parts: firstly, comparing the FX method with 3D elastic benchmark models featuring a complex defect, and, secondly, comparing it with measured data using a simpler machined defect.

## 2. Background

### 2.1. Guided Waves

**Figure 2:**
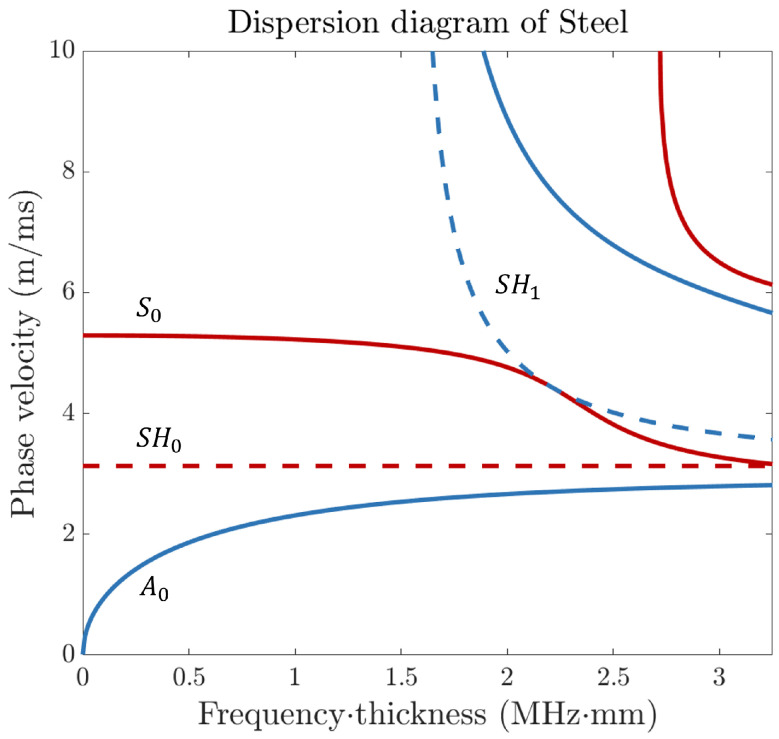
Dispersion curves for a steel plate. The phase velocity is plotted as a function of the frequency–thickness product for multiple wave modes. This study focuses on the $S_{0}$, $A_{0}$, and $SH_{1}$ modes.

Guided waves exhibit dispersive behaviour, characterised by the dependency of their phase velocity on frequency and thickness. This dispersion phenomenon is illustrated in Figure 2 for a steel plate.

While originally defined for flat plates, the negligible impact of wall curvature in thin-walled pipes allows for a reasonable approximation of Lamb wave behaviour. When propagated through pipelines, these waves follow helical paths, enabling the same mode to reach the receiver at different times (see Figure 1b). In guided wave tomography system design, mode selection impacts sensitivity to experimental uncertainties like noise and attenuation. Multiple dispersive Lamb modes in pipes of varying thickness complicate matters, causing interference at the receiver. Therefore, efforts usually prioritise lower frequencies, focusing on fundamental Lamb modes like the symmetric ($S_{0})$ and asymmetric ($A_{0})$ modes due to their manageable dispersion [3,27,43].

Recently, the utilisation of shear horizontal (SH) guided waves, particularly the $SH_{1}$ mode, has offered enhanced resolution compared to the fundamental Lamb modes [8]. However, the SH1 mode does not exist below the cut-off frequency, which, in the case of the steel plate in Figure 2, is at 1.6 MHz-mm. This limits the frequency range since increasing to higher frequencies would introduce higher-order modes. Due to the practical challenges of exciting them purely using omnidirectional transducers, higher-order Lamb modes are not considered. Instead, we focus on investigating the $S_{0}$, $A_{0}$, and $SH_{1}$ modes. Each of these modes has its advantages and disadvantages in tomography, as explained by previous authors [3,8]. In future research, we intend to use all three modes in a joint inverse scheme. Therefore, we need to validate the FX performance for all three modes against the elastodynamic case in our numerical studies. However, in our experiments, we only focus on the $A_{0}$ mode. This mode is the only one radiating into the air within the sensitive frequency range that we can record with our contact-free measurement set-up. This is due to its strong out-of-plane component.

The dispersion curves in Figure 2 form the basis for the velocity model used in wavefield extrapolation. Given a thickness profile, a wavefield is calculated for each frequency in the space–frequency domain. The time signal at any location can be reconstructed by multiplying the frequency domain wavefield data by the frequency domain source wavelet and applying an inverse Fourier transformation. This allows comparison with the synthetic data of the elastic model or experimental data. In this comparison, differences may still exist due to numerical errors. However, the crucial point that should emerge from this study is that these errors must be small compared to the defect response used to define misfits in the tomographic reconstruction.

### 2.2. Wavefield Extrapolation

For tomographic purposes, a variety of different forward models exists. The fastest among these is the ray-tracing technique utilising the high-frequency approximation of arrival times. Another approach to handling complex velocity models is employing a method operating within the frequency band rather than relying on a high-frequency approximation. The most comprehensive and accurate modelling methods fall under the category of computing the full wavefield using the two-way wave equation. Notably, the finite difference method [44] is applicable to both the acoustic and elastic two-way wave equations. The significant advantage lies in obtaining the entire wavefield alongside arrival times, facilitating its use in full waveform inversion. However, these two-way wave equation methods tend to be computationally demanding.

Alternatively, methods based on the one-way wave equation are often more computationally efficient compared to their two-way counterparts. These methods share similarities with seismic migration techniques. A one-way wavefield can be propagated in a homogeneous media using an extrapolation operator in the wavenumber domain [33]. Variations like Phase Shift Plus Interpolation [45] can address lateral velocity variations, though only approximately. To explicitly handle lateral velocity variations, multiplication in the wavenumber domain is substituted by spatial convolution in the space–frequency domain [35], as implemented by Holberg [36] and Blacquiere et al. [46]. This section serves as a detailed discussion of this method, referred to as the FX method, following Thorbecke et al. [47], since it is often unknown within the field of NDT. The tomography algorithm utilises the FX method alongside weighted least-squares operator optimisation. We have multiple reasons for its use. Firstly, the technique correctly handles frequency-dependent propagation effects, such as diffraction. However, the method models acoustic wave equations exclusively, without any elastic effects. Transmission effects and multiple scattering are not considered due to the one-way nature of the method. Secondly, in theory, it can handle propagation angles up to $90^{\circ }$. Still, in practice, the range is typically limited to around $60^{\circ }$. The weighted least-squares optimisation can decrease the operator size, boosting the computation speed. Finally, the implementation can be easily parallelised for each frequency.

To derive the FX method, let us consider the following acoustic wave equation in the frequency domain:

(1) $$\nabla^{2}P\left(\vec{r},\omega \right)+\frac{\omega^{2}}{c^{2}}P\left(\vec{r},\omega \right)=-S\left(\vec{r},\omega \right)$$

where $P(\vec{r},\omega )$ is the Fourier transform of the pressure field, $\vec{r}$ is the position vector $r=\sqrt{x^{2}+y^{2}+z^{2}}$, and *S* is the source term. Furthermore, ω is the angular frequency, and *c* is the propagation velocity. The FX method relies on Huygens’ principle, which states that a wave’s propagation through a medium can be understood by considering contributions from all secondary sources along its wavefront. Mathematically, this principle is formalised by the Kirchhoff–Helmholtz integral, which calculates the wavefield at a point within a volume *V* by integrating contributions from the wavefield and its normal derivative over the boundary surface Σ.

(2) $$P\left({\vec{r}}_{A},\omega \right)=-\oint_{\Sigma }\left(P\nabla G-G\nabla P\right)\cdot \vec{n}dS,\;{\vec{r}}_{A}\in V$$

The Green’s function *G* describes the wavefield due to a point source in *A* as follows:

(3) $$\begin{array}{c} \nabla^{2}G\left(\vec{r},{\vec{r}}_{A},\omega \right)+\frac{\omega^{2}}{c^{2}}G\left(\vec{r},{\vec{r}}_{A},\omega \right)=-\delta \left(\vec{r}-{\vec{r}}_{A}\right)\\ G\left(\vec{r},{\vec{r}}_{A},\omega \right)=\frac{e^{-i\omega \left|\vec{r}-{\vec{r}}_{A}\right|/c}}{4\pi \left|\vec{r}-{\vec{r}}_{A}\right|} \end{array}$$

#### Rayleigh II — Integral

**Figure 3:**
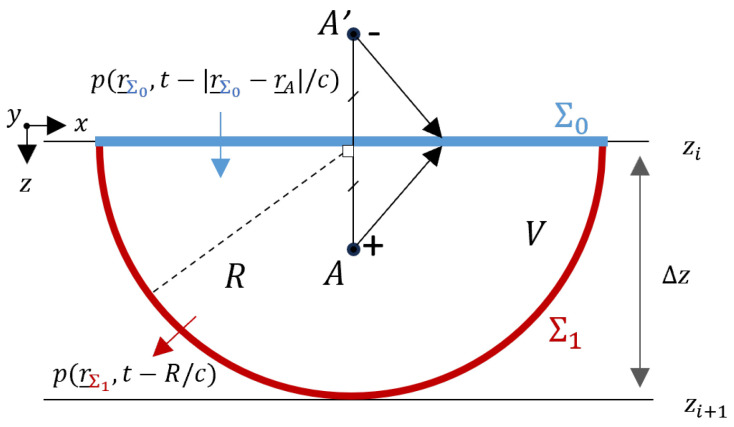
This configuration is used to derive the Rayleigh II integral. This integral describes the pressure field in the lower half-space as reconstructed by a dipole source at $z_{i}=z_{0}$.

In tomography, sources are often located on one side of an infinite flat plane $\Sigma_{0}$. Integrating $\Sigma_{0}$ into a closed surface Σ establishes a boundary where energy enters *V* solely through $\Sigma_{0}$ and exits through the closing part $\Sigma_{1}$ in the lower hemisphere (see Figure 3). Here, we focus on forward wavefield extrapolation away from the sources using the causal form of the Kirchhoff integral. Leveraging the knowledge that all sources are above an infinite flat plane achieves two objectives: avoids the need to measure along a closed surface and eliminates one term from the Kirchhoff integral. To eliminate the second term, $A^{\prime }$ is assumed to be a negative point source that forms the mirror image of A, rendering G=0 over $\Sigma_{0}$. Next, we can put $\Sigma_{1}$ at infinity and obtain the general formulation of the Rayleigh II integral.

(4) $$P\left({\vec{r}}_{A},\omega \right)=2\int_{\Sigma_{0}}P\left(\vec{r},\omega \right)\frac{\partial G}{\partial n}dS$$

When substituting the Green’s function from Equation (3), we obtain

(5) $$P\left({\vec{r}}_{A},\omega \right)=\frac{z_{A}-z_{0}}{2\pi }\int_{-\infty }^{\infty }\int_{-\infty }^{\infty }P\left(\vec{r},\omega \right)\frac{1+j\frac{\omega }{c}\Delta r}{\Delta r^{3}}e^{-j\frac{\omega }{c}\Delta r}dxdy$$

This representation corresponds to the one-way Rayleigh II integral. Now, we choose an arbitrary propagation distance $z_{i+1}$ for point A, as depicted in Figure 3. Note that, for the purpose of this theoretical derivation, we choose the propagation direction to be *z* in order to be consistent with the seismic literature. According to the Rayleigh II integral, a pressure field at the propagation level $z_{i+1}$ can be synthesised through a dipole distribution at level $z_{0}$, weighted with the pressure wavefield at level $z_{0}$ [41].

## 3. Extrapolation Operators

For each propagation level $z_{i}$, Equation (5) can now be rewritten as a convolution operation in Cartesian coordinates [48,49] as

(6) $$P\left(x,y,z_{i+1},\omega \right)=W\left(x,y,z_{i+1},z_{i},\omega \right)*P\left(x,y,z_{i},\omega \right)$$

with

(7) $$W\left(x,y,z_{i+1},z_{i},\omega \right)=\frac{\partial G\left(x,y,z_{i+1},z=z_{i},\omega \right)}{\partial z}$$

where $W\left(z_{i+1},z_{i}\right)$ is a propagation operator that describes propagation from level $z_{i}$ to level $z_{i+1}$. The convolution in the space–frequency domain corresponds to a multiplication in the wavenumber–frequency domain.

(8) $$\tilde{P}\left(k_{x},k_{y},z_{i+1},\omega \right)=\tilde{W}\left(k_{x},k_{y},z_{i+1},z_{i},\omega \right)\tilde{P}\left(k_{x},k_{y},z_{i},\omega \right)$$

where $k_{x}$ and $k_{y}$ are the lateral wavenumbers. The tilde symbol (∼) denotes the wavenumber–frequency domain. For a homogeneous medium, the extrapolation operator $\tilde{W}$ becomes

(9) $$\tilde{W}\left(k_{x},k_{y},z_{i+1},z_{i},\omega \right)=\exp\left(-jk_{z}\Delta z\right),$$

with

(10) $$k_{z}=\left\{\begin{array}{ccc} \sqrt{k^{2}-\left(k_{x}^{2}+k_{y}^{2}\right)} & \;\mathrm{for}\; & k_{x}^{2}+k_{y}^{2}\leq k^{2},\\ -j\sqrt{\left(k_{x}^{2}+k_{y}^{2}\right)-k^{2}} & \;\mathrm{for}\; & k_{x}^{2}+k_{y}^{2}>k^{2}, \end{array}\right.$$

where $k_{z}$ is the wavenumber in the propagation direction, and $\Delta z=z_{i+1}-z_{i}$ is the extrapolation distance. This is the phase-shift operator described by Gazdag [33]. Thus, in the homogeneous case, wavefield extrapolation from one propagation level to the next can be simply performed by multiplication with this phase-shift operator. Note that, for $\left(k_{x}^{2}+k_{y}^{2}\right)>k^{2}$, the wavefield becomes evanescent (i.e., exponentially decaying).

We have been examining the general formulation in 3D so far. However, since we are solving a 2D problem for an unwrapped pipe, we omit the term $k_{y}$ in Equation (9). The extrapolation operator for a 2D medium becomes

(11) $$\tilde{W}\left(k_{x},\Delta z,\omega \right)=\exp\left(-jk_{z}\Delta z\right).$$

This operator is only valid for a homogeneous medium. To allow for medium variations in the lateral direction, it is necessary to return to the space–frequency domain and perform the convolution operation. Convolution with a spatial operator is used at each spatial grid point and frequency component, assuming local homogeneity.

**Figure 4:**
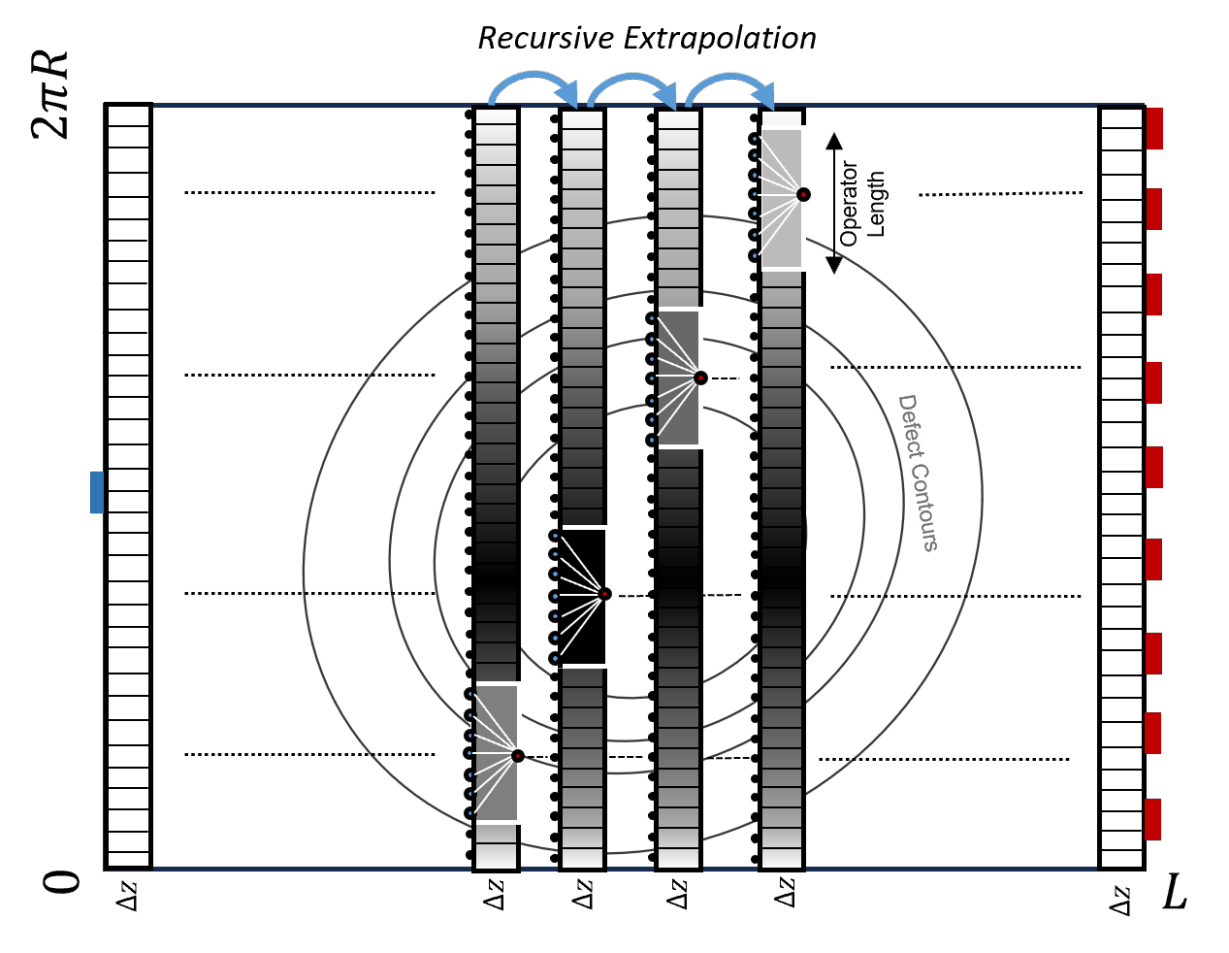
Recursive wavefield extrapolation, as per [47], uses a different operator for extrapolating the pressure field at every lateral position with varying velocities from one propagation level, *z*, to the next. A shorter operator is preferred due to the assumption of medium homogeneity within its length.

Figure 4 illustrates the use of space-variant convolution operators in recursive wavefield extrapolation within an unwrapped pipe. Based on the local velocity, another operator is used at every lateral position. To obtain the space–frequency expression, one can perform an inverse Fourier transform of Equation (9), which becomes a scaled Hankel function [35]:

(12) $$W\left(x,\Delta z,\omega \right)=-jk\frac{\Delta z}{2r}H_{1}^{\left(2\right)}\left(kr\right)$$

where $r=\sqrt{\left(x^{2}+\Delta z^{2}\right)}$ and $H_{1}^{\left(2\right)}\left(kr\right)=J_{1}\left(kr\right)-jY_{1}\left(kr\right)$, and $J_{1}$ and $Y_{1}$ are the first-order Bessel functions of the first and second kind, respectively.

Efficiency is a key consideration in the implementation of spatial convolution operators. Utilising the full-length operator is computationally expensive. Therefore, it is essential to seek the most efficient implementation method, often resulting in the creation of extrapolation operators that are as short as possible. This can be achieved by truncating the operators to a finite number of points in the space domain. The accuracy of these truncated operators is typically evaluated by comparing their wavenumber spectra to ensure they adequately preserve evanescent energy. The recursive application of explicit extrapolation operators from one level to the next underscores the importance of stability in their design. To ensure stability, it is advisable to limit all amplitudes of the operator in the wavenumber domain so that they do not exceed unity. This practice often leads to stable outcomes even in laterally inhomogeneous media scenarios. Numerous techniques have been developed to enhance the accuracy and efficiency of the FX method [36,37,38,39,40]. These methods can be extended for anisotropic media using Thomsen parameters [31,50,51,52]. We adopt the weighted least-squares method [47,53], which involves determining suitable weighting factors for components both within and outside the propagation region. This optimisation process provides an efficient and stable solution because it aims to construct a short convolution operator with a wavenumber spectrum closely resembling the exact formulation in a specified wavenumber range. This problem is typically formulated as

$$\begin{array}{cc} \tilde{W}\left(k_{x},\Delta z,\omega \right)= & \int_{x_{1}}^{x_{2}}\exp\left(jk_{x}x\right)W\left(x,\Delta z,\omega \right)dx\\ \; & \;\mathrm{for}\;k_{x,1}\leq k_{x}\leq k_{x,2}, \end{array}$$

where W(x,Δz,ω) is the operator to be determined. For more details on this optimisation procedure, the reader is referred to Thorbecke et al. [47]. The maximum propagation angle is an important input parameter for the operator optimisation. In our current validation study, we set the maximum allowable angle at 90 degrees. However, for longer pipelines being studied for tomography, this angle will be reduced to 60 degrees. This reduction in angle allows for a substantially shorter operator length. The tomographic algorithm allows the pre-calculation of operators, which are subsequently stored in a table for a range of wavenumbers [54]. This step is performed only once, and the calculated operators can be reused multiple times, making the method highly efficient for multiple source positions and inversion iterations.

## 4. Methods

### 4.1. Numerical Validation

For the numerical validation of the FX method, we utilise full three-dimensional elastodynamic simulation to fully capture the complexity of guided wave scattering. We employ two distinct methods for this purpose. Firstly, Finite Element Modelling (FEM) in the frequency domain using commercial software (COMSOL). This method enables realistic defect modelling through CAD by resolving complex geometries with unstructured meshes. We primarily employ FEM in the frequency domain to gain insight into the scattering pattern caused by the defect and to identify any mode conversions that occur. However, FEM faces scalability challenges, particularly when generating synthetic measurements for very large models and across an entire frequency bandwidth, which is crucial for testing tomography in future applications. To address this limitation, we also develop a finite difference method in the time domain (FDTD). The results obtained from the FDTD are used to evaluate the accuracy of the FX method in terms of arrival times and amplitudes.

#### 4.1.1. Finite Element Modelling in the Frequency Domain

**Figure 5:**
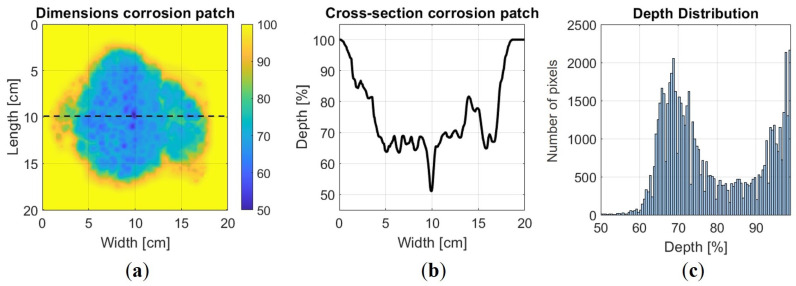
In numerical validation, a laser-scanned defect patch is used. (**a**) The dimensions of the defect shape. (**b**) A cross-sectional view along the deepest part of the defect. (**c**) Distribution of the wall loss values.

The FEM study considers corrosion patches in a 10 mm thick steel plate (z-direction) with material properties E=200 GPa, ν= 0.3, ρ = 7850 kg/m^3^. The plate measures 1.5 m (y-direction) in length and 0.6 m (x-direction) in width. From now on, the z-direction is reserved for sample thickness, and the wavefield propagates in the x–y direction, contrary to the theoretical derivation of the FX method in Section 2.2. The corrosion shape illustrated in Figure 5 is obtained via laser scanning with a minimum depth set at half the nominal thickness.

The model is replicated two times to account for helical modes n=−1,1 (as shown in Figure 1). Additionally, a perfectly matched layer (PML) boundary is implemented around the plate to mitigate reflections originating from its edges. For excitation, a source with a frequency of f=50,150,300 kHz is used for the $A_{0}$, $S_{0}$, and $SH_{1}$ modes, respectively. We utilise a combination of a tetrahedral mesh around the defect and the source and a hexahedral structured mesh for the remaining areas. For discretisation, we use quadratic shape functions. After conducting a convergence study, we find that 16 grid points per wavelength are required.

#### 4.1.2. Finite Difference Modelling in the Time Domain

**Figure 6:**
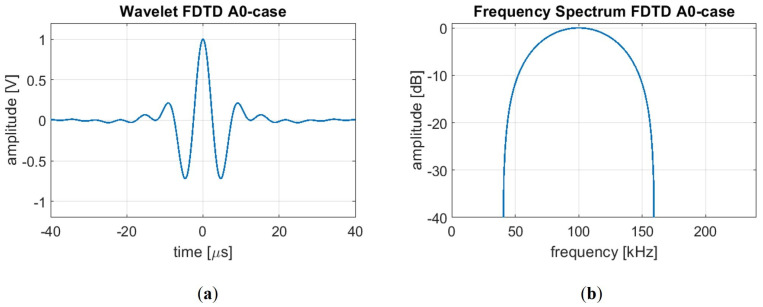
The cosine spectrum source wavelet for FDTD modelling of the $A_{0}$ excitation characterised by (**a**) its time domain representation obtained through an IFFT of the frequency spectrum, (**b**) the defined frequency spectrum with a centre frequency of 100 kHz and a bandwidth of 120 kHz.

Our 3D finite difference implementation relies on a rotated staggered grid. This grid structure allows us to simulate elastic wave propagation within a medium with free surfaces without applying explicit boundary conditions at the solid–vacuum interface [55,56]. Our investigation focuses on analysing a corrosion patch, depicted in Figure 5, embedded within a 10 mm thick steel plate. The corrosion patch’s maximum depth is set to cover half the nominal thickness for the $S_{0}$ and $A_{0}$ modes and 80% of the nominal thickness for the SH1 mode. For excitation, we define a cosine spectrum source wavelet with a centre frequency of 100 kHz for the $A_{0}$ mode, as shown in Figure 6, 180 kHz for the $S_{0}$ mode, and 250 kHz for the $SH_{1}$ mode and a bandwidth of 120 kHz. Also for this method, after a convergence study, it is found that 16 points per wavelength are required for meshing.

### 4.2. Mode Excitation

**Figure 7:**
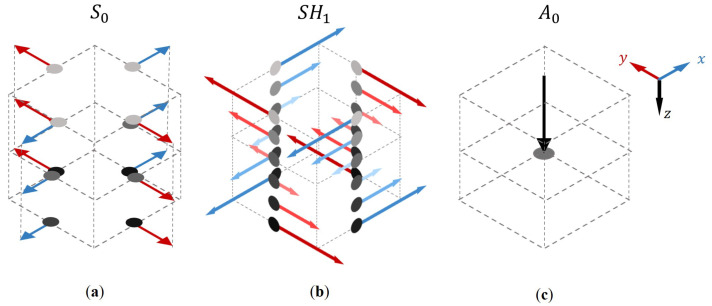
The excitation of a single-wave mode. (**a**) Excitation of the $S_{0}$ mode using four diverging vectors at each depth level. (**b**) Excitation of the $SH_{1}$ mode by rotating vectors along a thickness level. Each node is weighted with a calculated displacement curve through the thickness. (**c**) Excitation of the $A_{0}$ mode using a perpendicular vector on the centre line. In order to create a dipole, these excitations are doubled at a grid point distance with opposite signs in all simulations.

A challenge in 3D elastodynamic simulations is exciting pure single-wave modes. This is relatively straightforward for the $A_{0}$ mode. We only need a single node on the centre line of the plate where we excite a normal force for finite element (FEM) simulations and excite a velocity node in the z-direction for finite difference time domain (FDTD) simulations. Exciting the $S_{0}$ and $SH_{1}$ modes is more complex, so we employ a linear combination of point sources in the x- and y-direction. The excitation nodes are illustrated in Figure 7. In the FEM simulation, the $S_{0}$ mode is excited on the centre line as a radially divergent excitation. In contrast, the $SH_{1}$ mode is excited on the top and bottom as a torsional excitation in opposite directions. Nodes are excited throughout the entire thickness profile to excite the modes in FDTD simulations effectively. For the $SH_{1}$ mode, the through-thickness displacement curves are calculated. Subsequently, all nodes of excitation at each thickness level are coupled with the respective mode displacement. For both the FE and FD implementations, we excite a secondary source at a grid point distance with all vectors in the opposite direction. This is to simulate an equivalent source to the FX method, which effectively acts as a dipole, as described by the Rayleigh II integral (Equation (5)). On the receiver side, we receive the x, y, and z components of the velocity of the wavefield. For the $A_{0}$ mode, we examine the z component. All received components of the wavefield for the $S_{0}$ and $SH_{1}$ excitations are decomposed into their curl- and divergence-free potentials, representing the radial and transverse wavefield components, respectively. These are then compared with the FX wavefield.

### 4.3. Experimental Set-Up

**Figure 8:**
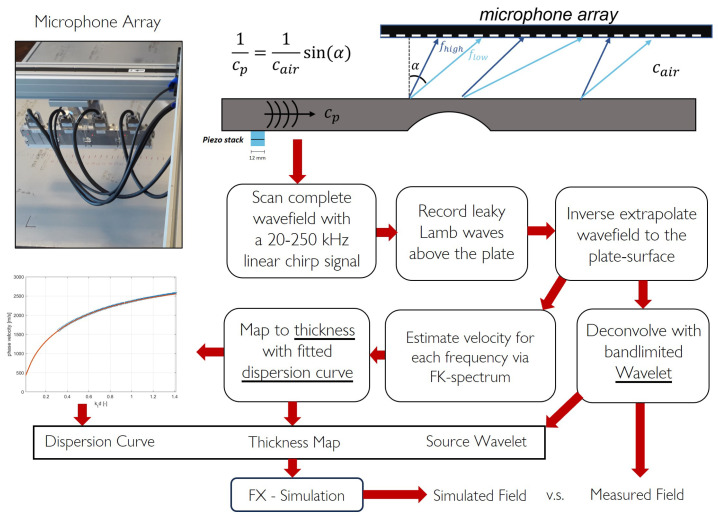
Use of a microphone array at a 20 cm distance to scan the full wavefield. By backpropagating the wavefield, we retrieve it back at the plate’s surface. Using the DVM technique, we can reconstruct a thickness map and a dispersion curve. This, along with a selected source wavelet, forms the input data for the FX model.

The experimental validation is conducted using an 8 mm thick aluminium sample containing a machined defect measuring 130 mm in diameter and reaching a maximum depth of 6.7 mm. The machined defect intentionally extends to a depth exceeding 80% of the nominal wall thickness, aiming to challenge the FX method by introducing high velocity gradients. For the measurement of the full wavefield, a contact-free inspection technique is utilised, the details of which are described by Volker et al. [57]. A schematic overview of the acquisition workflow is shown in Figure 8.

**Figure 9:**
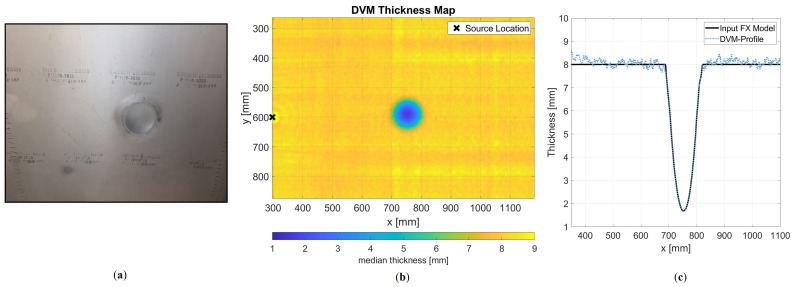
(**a**) Aluminium specimen with the machined defect. (**b**) Reconstructed thickness map with the DVM technique. (**c**) The thickness profile used for the model validation is indicated in black.

Leaky Lamb waves are detected with a microphone array at a 20 cm stand-off distance from the plate surface. Any Lamb wave mode can be employed, provided it exhibits sufficient out-of-plane displacement to generate a strong signal in the air. Here, we focus solely on the $A_{0}$ mode. The source transducer consists of two piezo elements stacked together, each 12 mm in diameter and 2 mm thick, with the active sides facing each other and the grounds on the outside. At the bottom of the plate, the transducer is driven with a 2 ms linear chirp signal to enhance the signal-to-noise ratio (SNR). This eliminates the need for additional measurement averaging. The microphone array, comprising 128 MEMS sensors, has a sensor spacing of 3 mm and spans 384 mm in total length. The scanning system operates in an interleaved mode to achieve a spatial sampling of 1 mm. Measuring at a non-zero stand-off distance introduces complexity due to the frequency dependence of the refraction angle in accordance with Snell’s law. To address this, the measured wavefield is backpropagated to the surface, eliminating the refraction effect. This backpropagation, achieved through inverse wavefield extrapolation in the wavenumber–frequency domain as detailed in Equation (9) in Section 2, calculates the wavefield as if measured directly at the plate’s surface. After backpropagation, the surface wavefield of the aluminium plate can be used to retrieve the local wave velocity for specific frequencies. Termed Direct Velocity Mapping (DVM) [57], this approach converts the wavefield into a velocity map, similarly to techniques like estimating local wavenumbers for laser Doppler vibrometer measurements [58,59]. A local phase velocity for each frequency component is determined by measuring local wavenumbers at different frequency slices from the data cube. A dispersion curve is estimated from the measurements using the known nominal thickness. The local thickness is obtained by mapping phase velocity to thickness. Each frequency component provides a thickness measurement which is then averaged to provide the thickness map of the sample, as shown in Figure 9. The obtained thickness map and the calculated dispersion curve create the input velocity model for the FX simulation. Also, for the simulation, we define a cosine spectrum source wavelet with a central frequency of 120 kHz and a bandwidth of 100 kHz. Finally, we perform wavelet shaping on the backpropagated measured wavefield by means of deconvolution with this source wavelet. This allows us to directly compare the modelled data with the measured data.

## 5. Results

### 5.1. Frequency Domain Modelling

**Figure 10:**
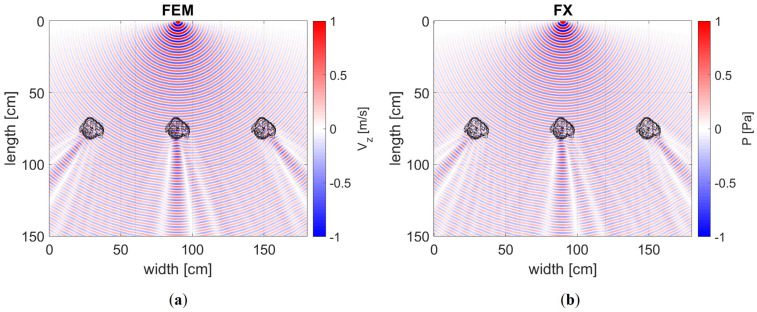
Real part of the complex wavefield of an $A_{0}$ mode excitation at 50 kHz showing the direct path and two helical modes. (**a**) The out-of-plane velocity component for the 3D elastic model using a dipole source to ensure an equal directivity with the FX method. (**b**) The pressure level for the 2D acoustic model. Note that both the velocity and pressure scale linearly, and, although we are looking at different physical quantities, this does not affect the relative (normalised) amplitude and phase differences used in tomography.

**Figure 11:**
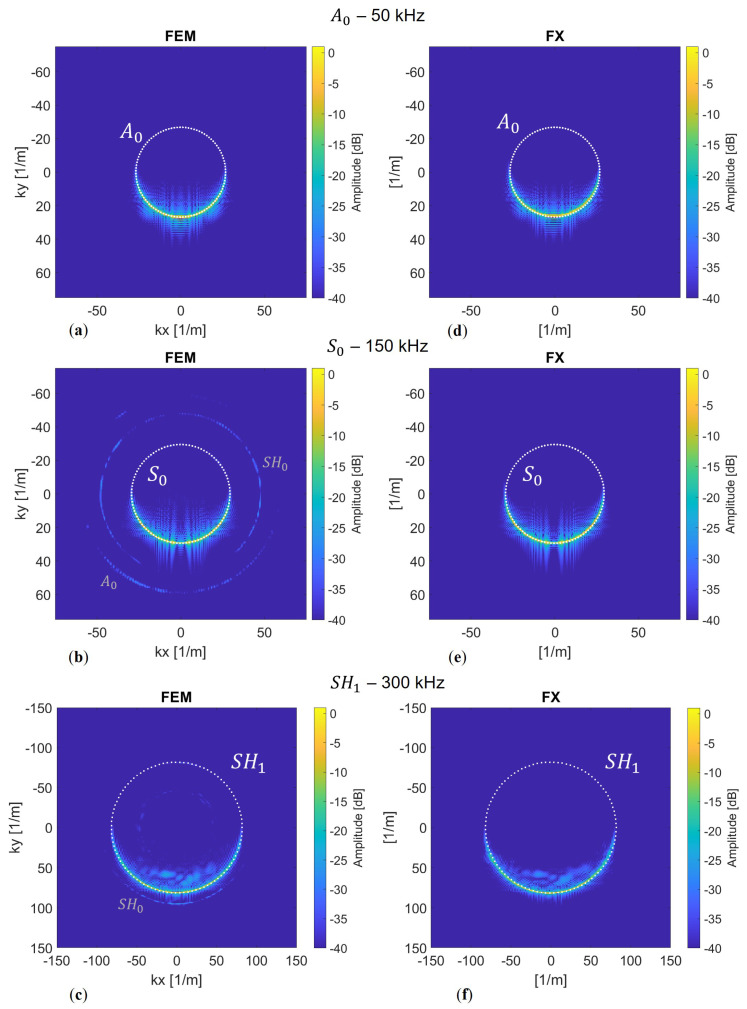
Wavenumber spectra of both the FE method and FX model. (**a**) The $A_{0}$ mode does not show any mode conversions or backscatter. In (**b**), the $S_{0}$ mode converts into the $SH_{0}$ and $A_{0}$ mode, but with very low amplitudes. In (**c**), the $SH_{1}$ mode shows some minor mode conversion into the $SH_{0}$ mode due to the defect. (**d**–**f**) The spectra for the FX method exhibit the same scattering profiles minus the mode conversions.

After performing FE modelling, our aim is to examine the scattering and diffraction pattern resulting from the realistic defect that is illustrated in Figure 5. In Figure 10, we present the monochromatic wavefield at 50 kHz, including the first-order helical modes for an $A_{0}$ source positioned at the centre of the domain. This is similar to Figure 1b, where we display three times 2πR (60 cm), resulting in a total width of 180 cm. The amplitudes are normalised between minus one and one. A notable observation from the visualisation is the strikingly similar diffraction pattern characterised by the focusing of energy behind the defect. This observation is consistent with our theoretical expectations for the $A_{0}$ mode. This is because the presence of the defect induces a decrease in phase velocity, as depicted in Figure 2. What is not evident in the wavefield is the presence of backscatter or reflections. This is because of the one-way wave equation modelling method used in this approach. In contrast, the elastic FE model does consider backscatter, but the relatively smooth corrosion defect does not create enough medium contrast for reflection. Additionally, there is no sign of any mode conversion. To assess the scattering in more detail, we analyse the wavefield in the wavenumber–frequency domain using a spatial 2D Fourier transformation, as shown in Figure 11. In the wavenumber domain, the expected velocity for the excited mode is observed. The amplitude of the excited mode is normalised to 1, allowing other present modes to be read as relative amplitudes in decibels. In the figure for the $A_{0}$ mode, the imprint of the three defects at angles of [−45 0 45] degrees from the origin is visible for both modelling schemes. No energy is observed in the negative wave numbers in the $k_{y}$-direction, indicating no backscatter. Also, no other wave modes are present. Similarly, for the $S_{0}$ and $SH_{1}$ excitation models, no backscatter is observed for the primary excited mode. However, for the $S_{0}$ mode, two other modes emerge in the elastic model, still with very weak amplitudes.

The mode-converted $A_{0}$ mode propagates in the positive direction with an amplitude of approximately −40 dB. The $SH_{0}$ mode-converted wave with an amplitude of −30 dB is only slightly stronger. However, it propagates laterally, which means it does not affect our pipe experiments. This is because we take measurements up to an angle of approximately 60 degrees at the farthest receiver location from the source. The $SH_{1}$ excited mode also excites other modes; only the presence of the $SH_{0}$ mode is visible at −30 dB. At this frequency, the SH1 mode shows conversion to the $A_{1}$ mode but with an amplitude of −50 dB, indicating a minimal signal strength.

### 5.2. Arrival Time and Amplitude Differences

**Figure 12:**
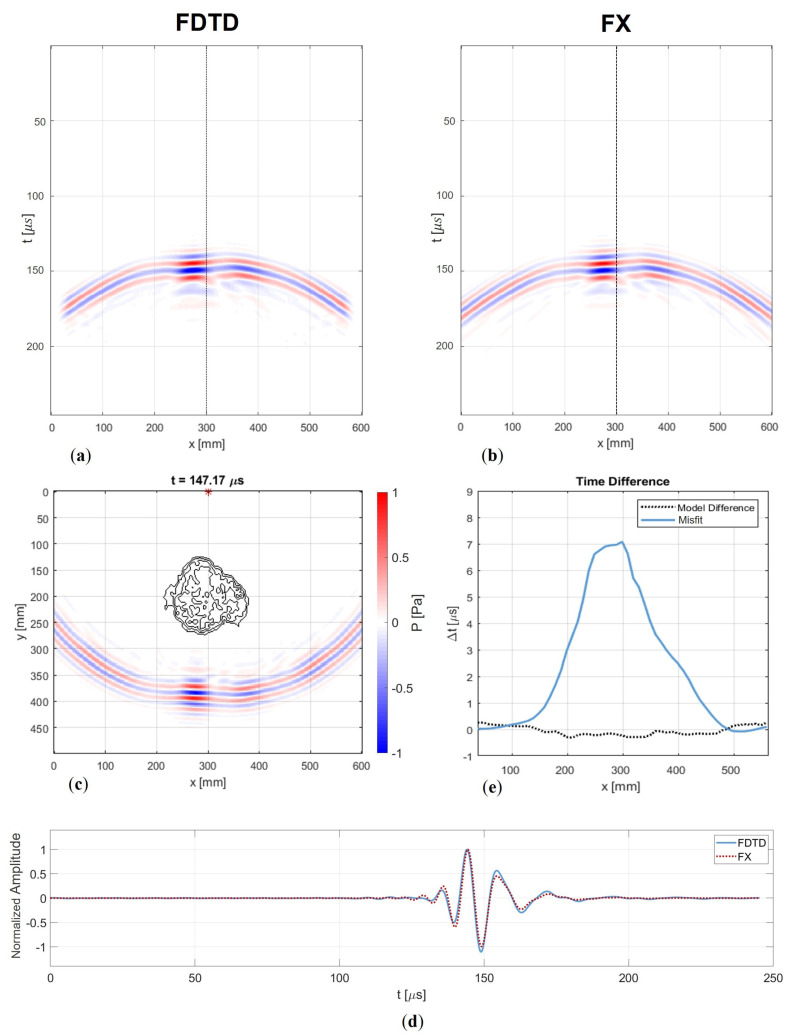
Similarity between modelling methods. (**a**) FDTD wavefield recorded at a receiver line at y=400 mm with tapered edges. (**b**) FX wavefield recorded at y=400 mm. (**c**) Snapshot of the FX wavefield showing the energy focusing of the $A_{0}$ mode behind the defect. (**d**) Time trace for both methods at y=400 mm and x=300 mm. (**e**) Shown by the black dashed line is the time difference between the two records (**a**,**b**), and, in blue, for reference, is the difference between a modelled pristine case and the defect case.

Now that we have observed that our model adequately describes the physics for tomographic purposes, we aim to quantitatively determine whether the differences between an elastic model and our acoustic implementation are sufficiently small in both amplitude and phase. In Figure 12, we present the results of the elastic finite difference simulations on the rotated scattered grid compared to the FX method.

Again, we observe the energy focusing behind the defect in (a). When we select a receiver line at 400 mm, we obtain (a & b), and, when we take a trace aligned with the source, we obtain Figure 12d. The two recorded wavelets are almost identical, with slightly more significant differences in amplitude than in phase. To assess accuracy more comprehensively, it is interesting to observe changes with offset. Therefore, we perform a cross-correlation on the corresponding records in (a) and (b). We then identify the maxima for each offset to determine the time differences at each receiver position. These arrival time differences are represented by the black dashed line in Figure 12e. Noticeably, there is slightly more fluctuation around the focal point of the defect position between *x* = 200 and *x* = 300 mm. Moreover, with larger offsets, the differences tend to increase somewhat. This aligns with our observation of accumulating numerical dispersion, particularly from the FD method. In order to assess whether the difference between FX and 3D modelling is acceptable, a comparison is made with a defect-free response. The requirement is that the differences between modelling schemes should be significantly smaller than between a defect and a defect-free response. The latter difference is called the misfit.

**Figure 13:**
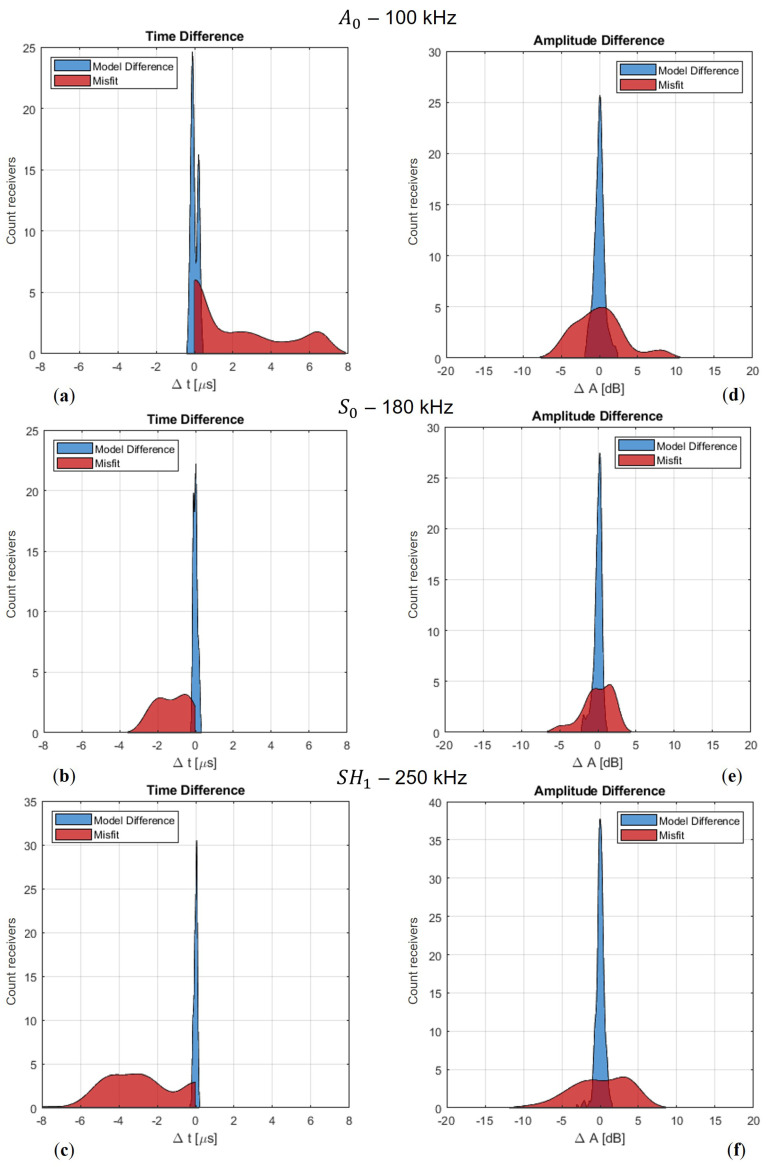
For different wave modes, the distribution of the model errors is shown in blue, and the misfit between the pristine and defect cases is shown in red. (**a**–**c**) Arrival time differences in microseconds; (**d**–**f**) amplitude ratios in decibels.

To illustrate the differences between the models in contrast to the misfit, we choose to plot all the differences as distributions, as shown in Figure 13. Upon examining the figure, we can observe that the distributions of the model differences have a mean of zero, which indicates there is no bias. Furthermore, we notice that the width is significantly smaller in comparison to the misfit. For instance, if we consider the time differences for the $A_{0}$ mode, we can see that the model error, represented in blue, is approximately 0.25 μs. In contrast, the misfit time difference, depicted in red, amounts to a maximum of 7 μs. While there is a disparity between the 2D FX model and the benchmark 3D FDTD, indicating an error, it is much smaller than the misfit. The question remains: what kind of misfit is needed to push the optimisation problem in the right direction and converge towards the actual defect? To this end, we conduct some preliminary tests with our frequency domain tomography algorithm, and these initial tests show that we need a minimum phase angle difference of approximately 0.35 radians. In comparison to the misfit distributions shown in Figure 13, at least an arrival time difference of approximately 0.5, 0.3, and 0.2 μs is required for the S0, A0, and SH1 modes, respectively, at the given centre frequencies. The analysis can also be conducted for the amplitudes, as depicted in (d–f). Here, we take the absolute values of the wavefield at the centre frequency and examine the ratio between the two. In terms of amplitude, the absolute model error is approximately 2 dB, whereas the misfit can reach up to 8 dB. We repeat this analysis for the $S_{0}$ and $SH_{1}$ modes, although, for the $SH_{1}$ mode, we limit the maximum wall thickness loss to 20% instead of 50%. We observe similar model errors for the modes. For the $SH_{1}$ mode, we anticipate comparable performance given the significant time and amplitude misfits. However, due to the lower sensitivity of the $S_{0}$ mode, as inferred from the dispersion curve gradient in Figure 2, the spread of time and amplitude differences is notably smaller. Therefore, this mode will be more affected by the model error as well as other forms of noise. Adding the amplitude information for the $S_{0}$ mode to the total misfit function appears to provide limited value.

### 5.3. Experimental Results

**Figure 14:**
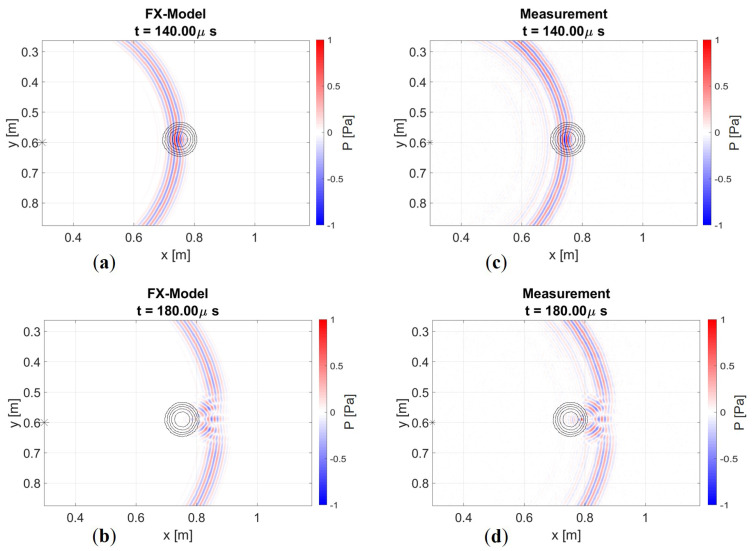
Snaphots of modelled and measured wavefield: (**a**–**c**) at t=140μs, observing a focusing effect while passing the defect; and (**b**–**d**) at t=180μs diffraction after the defect, showcasing a similar interference pattern. At the microphone array, we measure a pressure level. Similarly, the modelled wavefield also represents pressure.

We have confirmed through numerical simulations that the FX method is an adequate way to describe the physics of guided waves. Now, we aim to demonstrate this through measured data. Figure 14 shows two snapshots that compare the two models. The first snapshot captures the moment of encountering the defect, while the second one shows the diffraction pattern after the wave has passed through the defect.

**Figure 15:**
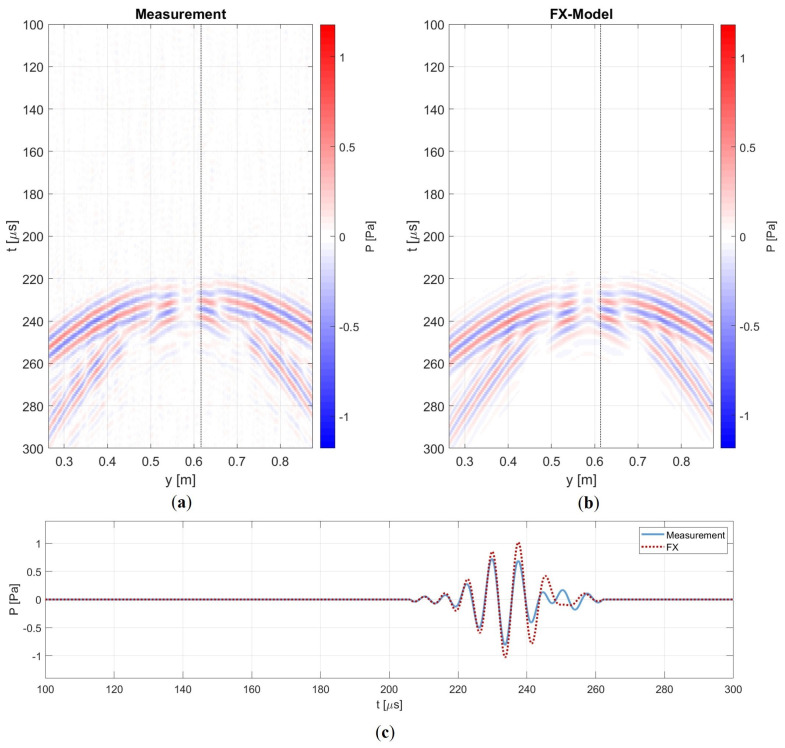
Single shot gather with a receiver line at x=1 m for both the measurement (**a**) and the FX model (**b**), showcasing a similar interference pattern. (**c**) Time trace at *x* = 1 m and *y* = 0.62 m. The measured wavefield exhibits lower amplitude, primarily due to source directivity. Additionally, the tail of the measured wavelet is influenced by a source ringing effect.

Some minor differences can be observed. A faint source ringing imprint is still discernible in the measurement, which can be filtered out. Additionally, for the representation of the amplitude in the snapshot at 140 μs, we apply a weighting of $1/\sqrt{D_{rel}}$, where $D_{rel}$ represents the relative thickness at the defect position. This adjustment accounts for the increased out-of-plane displacement resulting from the thinning of the plate in the measurement, which is not captured in a 2D model. However, this discrepancy is negligible for wavefronts measured at a receiver line behind the defect. Furthermore, the interaction with the defect and its associated diffraction pattern seems identical. Once again, no backscattering or mode conversion can be observed. Thus, these results are consistent with the expectation of the numerical experiment. When we extract a receiver line at x=1 m, we obtain Figure 15.

We observe a resemblance in the interference pattern, and the arrival times are nearly identical. However, upon closer examination of the time trace, we notice that, initially, the phases of the wavelets neatly overlap. Around 250 μs, however, the ringing effect begins to interfere with the wavelet. Additionally, there is a difference in amplitude, most likely stemming from the difference in source directivity. Specifically, the modelled dipole differs from the measured monopole, with the dipole concentrating its energy more along the axial propagation line. Consequently, for these small offsets, we observe a higher amplitude with the FX method.

## 6. Conclusions

This paper presents the recursive wavefield extrapolation method and its modelling performance for further use in corrosion mapping. Our 2D acoustic model (FX) simulates the behaviour of $A_{0}$, S0, and $SH_{1}$ waves interacting with defects. To obtain a numerical benchmark solution of the full 3D elastic wave equation, we used commercially available finite element (FE) software and an in-house finite difference (FDTD) code based on a rotated staggered grid. Both the elastic and acoustic numerical methods produced highly consistent results. Through numerical and experimental testing, we confirmed that the results had no significant backscatter and minimal mode conversions. We evaluated both the time and amplitude differences between the elastic and acoustic methods, which are useful for a full waveform inversion implementation with a parameterised defect. The results also show which modes are relevant for considering amplitudes in the joint inverse scheme. Given the model errors, it became clear that including the amplitude information in the misfit for the $S_{0}$ mode will not contribute significantly to the convergence of the inverse problem. Our findings affirm the viability of our one-way acoustic model for tomographic applications, emphasising its efficiency and accuracy. Finally, the measured results also indicate that source directivity is significant for amplitude accuracy. Therefore, the correct directivity of transducers should be included in the model. Depending on the transducers used in the tomographic set-up, this can be achieved with the FX method by employing a combination of multiple point source positions. Further improvements can be made by extending the method to address anisotropic cases and accommodate complex geometries such as pipe bends. By enhancing this methodology, we aim to improve corrosion mapping capabilities, providing more accurate and comprehensive insights into pipeline integrity.

## Data Availability

The authors can provide the measurement data upon request.
